# Wellbeing intervention for chronic kidney disease (WICKD): a randomised controlled trial study protocol

**DOI:** 10.1186/s40359-018-0264-x

**Published:** 2019-01-08

**Authors:** Kylie M. Dingwall, Tricia Nagel, Jaquelyne T. Hughes, David J. Kavanagh, Alan Cass, Kirsten Howard, Michelle Sweet, Sarah Brown, Cherian Sajiv, Sandawana W. Majoni

**Affiliations:** 1 Menzies School of Health Research, Institute of Advanced Studies, Charles Darwin University, PO Box 4066, Alice Springs, NT 0870 Australia; 2 Menzies School of Health Research, Institute of Advanced Studies, Charles Darwin University, Darwin, NT 0811 Australia; 3 Division of Medicine, Royal Darwin Hospital, Darwin, NT 0811 Australia; 4 Centre for Children’s Health Research, Institute of Health & Biomedical Innovation and School of Psychology & Counselling, Faculty of Health, Queensland University of Technology (QUT), Brisbane, QLD 4101 Australia; 5 Sydney School of Public Health, Faculty of Medicine and Health, University of Sydney, Sydney, NSW 2006 Australia; 6 Western Desert Nganampa Walytija Palyantjaku Tjutaku, Alice Springs, NT 0870 Australia; 7 Central Australian Renal Services, Alice Springs Hospital, Northern Territory Department of Health, Alice Springs, NT 0870 Australia; 8 Top End Renal Services, Royal Darwin Hospital, Northern Territory Department of Health, Darwin, NT 0810 Australia; 9 Northern Territory Medical Program, Flinders University, Darwin, NT 0815 Australia; 10 Flinders University, Adelaide, SA 5042 Australia

**Keywords:** Renal, E-mental health, Indigenous, Wellbeing, Kidney disease

## Abstract

**Background:**

Incidence of end stage kidney disease (ESKD) for Indigenous Australians is especially high in remote and very remote areas of Australia (18 and 20 times the rate of comparable non-Indigenous people). Relocating away from family and country for treatment, adjusting to life with a chronic condition and time lost to dialysis cause grief and sadness which have immense impact on quality of life and challenges treatment adherence. We describe the first randomised controlled trial to address both chronic disease and mental health in Indigenous people with ESKD, which is the first to test the effectiveness of a culturally adapted e-mental health intervention in this population. It builds on an existing program of mental health research with demonstrated efficacy – the Aboriginal and Islander Mental Health Initiative (AIMhi) – to test the newly developed electronic motivational care planning (MCP) therapy – the AIMhi Stay Strong App.

**Methods:**

This is a 3-arm, waitlist, single-blind randomised controlled trial testing the efficacy of the Stay Strong App in improving psychological distress, depressive symptoms, quality of life and treatment adherence among Indigenous clients undergoing haemodialysis for ESKD in Alice Springs and Darwin with follow up over two periods of 3 months (total of 6 months observation). The study compares the efficacy of MCP using the AIMhi Stay Strong App with two control groups (control app intervention and treatment as usual) on participant-reported psychological distress (the primary outcome) using the Kessler Distress Scale (K10); depressive symptoms using the adapted Patient Health Questionnaire (PHQ-9); quality of life using the EuroQoL instrument (EQ5D) and adherence to dialysis treatment planning through file audit. Participants are randomised to receive MCP either at baseline (early treatment) or after 3 months (delayed treatment). The study also examines the cost effectiveness of this therapy in this setting through examination of health care service utilisation across groups during the first 3 months.

**Discussion:**

This project will contribute much needed evidence on the efficacy of an electronic wellbeing intervention for Indigenous people with ESKD – a group in which distress is likely to be unacceptably high, yet relatively untreated.

**Trial registration:**

Australian New Zealand Clinical Trial Registry; ACTRN12617000249358; Date registered: 17/02/2017.

## Background

The incidence of end stage kidney disease (ESKD) for Indigenous Australians in the Northern Territory (NT) is 15.3 times the rate of non-Indigenous Australians, with the burden of the disease on the increase (96% increase in Indigenous incidence nationally over the 1991–2010 period) [1]. People with ESKD require renal replacement therapy (regular dialysis or kidney transplant) to survive. Such treatments are resource intensive adding to an already high burden for the individual and health care system.

People with chronic kidney disease (CKD) sustain many losses - physical functions, cognitive abilities, and role in the family and workplace [2], and depression is common in those undergoing dialysis (25% with depressive symptoms when assessed by clinical interview, 40% when assessed by self-report measures) [3]. These levels are unacceptably high given that depressive symptoms are a risk factor for poor outcome in people with ESKD on dialysis [4].

Early intervention for psychological distress among people with CKD has the potential to prevent or defer onset of chronic and debilitating mental disorders and minimise the impact of wellbeing concerns on adherence and treatment outcomes. Meta-analyses estimate reductions in incidence of depression of approximately 20% when preventative interventions are delivered to the general population with no diagnosed depression at baseline [5, 6].

Despite recognition that psychosocial factors are associated with morbidity and mortality in many chronic conditions, including CKD, well-designed intervention studies are lacking [2, 4]. The most recent Cochrane Review (2005) failed to identify any randomised controlled trials (RCTs) assessing psychosocial interventions for depressed people on dialysis [7]. Furthermore, there is a significant lack of rigorous effectiveness trials for mental health interventions in an Indigenous context generally [8]. Given the scarcity of evidence, RCTs in this area are desperately needed [4].

One of the very few formally evaluated, culturally-adapted, mental health and chronic disease self-management interventions for Indigenous people was developed through the Aboriginal and Islander Mental Health Initiative (AIMhi). Assessment, psycho-education, and care-planning resources were developed following extensive consultation and collaboration with local Aboriginal mental health workers (AMHW) through exploration of local Indigenous perspectives of mental health and with recognition of the holistic nature of wellbeing [9–11]. ‘Motivational care planning’ (MCP) combines problem solving therapy and motivational interviewing, to create a ‘low-intensity’ treatment that differs from established approaches by utilising a holistic, strengths-based approach with pictorial tools [10, 12]. It was evaluated in one of the first successful NHMRC-funded RCTs assessing mental health interventions in a remote Indigenous context, with further qualitative studies and evaluations confirming acceptability and feasibility [9, 11, 13, 14]. The RCT showed that the MCP intervention resulted in significant improvements in well-being, life skills, and alcohol dependence among Indigenous clients with chronic mental illness, with changes sustained over 18 months [9].

MCP is a theoretically sound, evidence-based approach to comorbidity, which has been used in settings other than mental health including substance misuse, gambling and chronic disease self-management [13–15]. The therapy adopts an empowering, person-centred, holistic and strengths based perspective which acknowledges and promotes Indigenous cultural and family values and client self-management [16]. The AIMhi MCP intervention has recently been translated into a digital (tablet) format (the AIMhi Stay Strong App), making it even more interactive and visually appealing. The result is the first available e-mental health approach developed specifically for Indigenous Australians.

This study examines the 3- and 6-month impact and cost effectiveness of the MCP therapy delivered by the AIMhi Stay Strong App for improving mental health and wellbeing in a renal dialysis setting, relative to two control conditions.

## Methods

### Aims

The primary aim of the study is to determine whether MCP using the AIMhi Stay Strong App reduces psychological distress for Indigenous people receiving haemodialysis, relative to delayed-treatment control groups at 3 months, and whether benefits are maintained at 6 months post-recruitment. Secondary aims test the impact of MCP using the AIMhi Stay Strong App in improving depressive symptoms, Quality of Life (QoL) and dialysis treatment adherence. We also aim to examine the cost effectiveness of this therapy.

We hypothesise that MCP therapy using the AIMhi Stay Strong App will be cost-effective and superior to both a contact control using another app (Hep B Story) [17] and usual care, in reducing psychological distress and depressive symptoms, and improving quality of life and dialysis treatment adherence at 3 months. We expect that MCP that is received in the control groups after the 3-month assessment will result in those groups showing improvements in these outcomes between 3 and 6 months.

### Study design

**Fig. 1:**
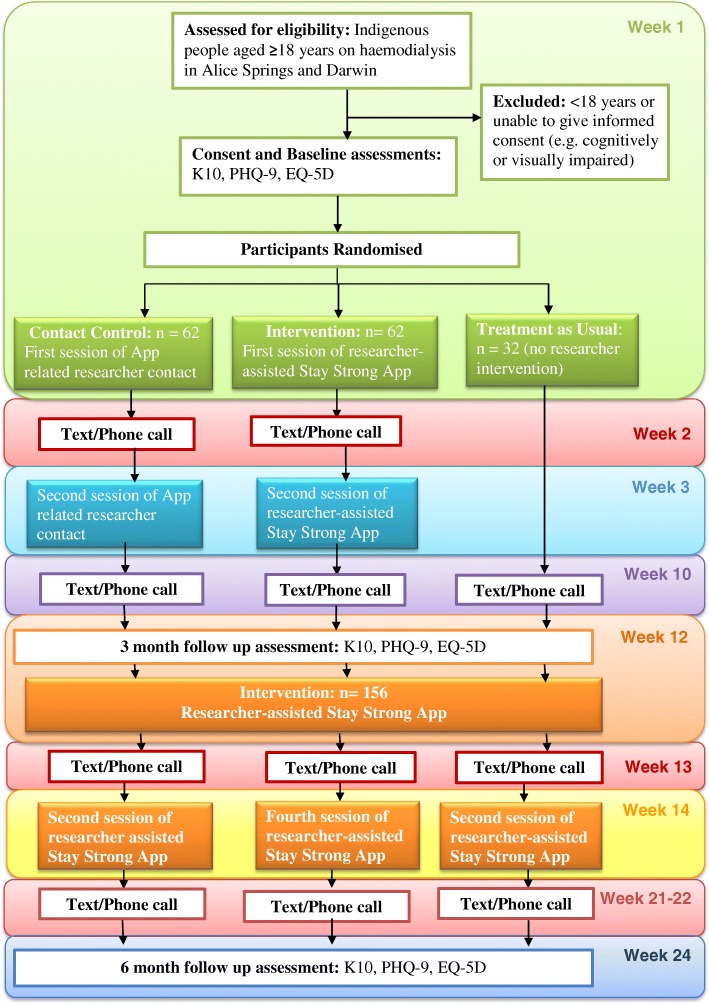
Participant Flowchart

This is a 3-arm, waitlist, single-blind randomised controlled trial testing the efficacy of the Stay Strong App MCP intervention in improving wellbeing among Indigenous clients undergoing haemodialysis for ESKD in Alice Springs and Darwin, with assessments at Baseline, 3 and 6 months (see Fig. 1 for participant flow). The three treatment conditions are: 1) Early treatment with MCP using the Stay Strong App 2) Contact control/Delayed treatment with the Stay Strong App (i.e. patients are engaged with the researcher for a similar time using an electronic application addressing general health issues) and 3) Treatment as usual/Delayed treatment with the Stay Strong App (see Fig. 1).

### Participants and setting

Indigenous people presenting to participating haemodialysis services in Alice Springs and Darwin – Western Desert Nganampa Walytja Palyantjaku Tjutaku (WDNWPT), Central Australian Renal Services, and Top End Renal Services, are approached to provide informed consent. Approximately 80% of participants are expected to speak English as a second or third language. Inclusion criteria include identification as an Aboriginal and/or Torres Strait Islander person and aged ≥18 years, currently receiving maintenance haemodialysis in Alice Springs or Darwin and having been receiving this treatment for more than 6 months. Exclusion Criteria are aged < 18 years, guardianship order in place, or inability to provide informed consent (e.g. because of cognitive or visual impairment). Whether potential participants meet criteria for inclusion is determined through liaison between project staff and the patient’s care coordinator.

### Consent and culturally appropriate approach

Research officers use pictorial information sheets and flipcharts, screening and intervention tools which use plain English and are available in 11 NT Aboriginal languages, to assist understanding. Participants are offered communication support (interpreters, translators, language recordings). Demographic information and outcome measures are collected using a tablet device that includes pictorial prompts and Aboriginal language recordings for each item (choice of 11 NT languages). Interpreters are also utilised where necessary. Assessment and treatment sessions occur at a place that is identified by the participant as most comfortable for them, which may be outdoors, at the clinic, while receiving dialysis, or at their accommodation.

### Randomisation

Eligible participants are randomised following baseline assessment using a block sequential random number sequence and an envelope system of randomisation, stratified by site, level of psychological distress (high or low) and access to respite dialysis in home community. An independent statistician created the allocation schedule with a computerized random number generator and investigators are blind to this schedule. Participants are allocated to Early Treatment with MCP using the AIMhi App, Contact Control/Delayed treatment or Treatment as usual/Delayed treatment at a ratio of 1:1:0.5. Participants are assigned a Study ID following randomisation to ensure confidentiality.

### Interventions

In addition to their allocated treatment below, all participants receive usual care from their renal service provider. The nature and extent of this care is monitored through file audits, to ensure no systematic difference between the treatment groups. Usual care is carried out according to the norms prevailing in the renal service and is informed by the needs of the client. Clinical files for each participant are carefully reviewed to examine the degree and nature of referrals or other treatment accessed over the study duration.

Either a phone (with credit) or $30 phone credit voucher is given to participants after completion of the baseline assessment and at each follow up assessment. All participants receive a text message or phone call at 10–11 weeks and 21–22 weeks post-baseline to remind them of the 3-month and 6-month assessments respectively (see Fig. 1).

#### Early treatment with MCP using the AIMhi stay strong app

##### Baseline

Participants who are randomised to the intervention complete a MCP interview of approximately 20 min duration using the AIMhi Stay Strong App (on a tablet device) at baseline, with a second 20 min session using the App within two to 4 weeks. Session 1 explores family, current strengths and worries. Participants are encouraged to set 1–2 goals that are achievable, meaningful, and practical for addressing an identified worry. The client-centred nature of the intervention ensures the goals are client driven and empowers them to take manageable steps toward achieving that goal. Session 2 reviews and refines the goals (identifies whether goals and steps were achieved) and helps clients address any barriers to goal attainment and set new goals as appropriate. Participants receive a text message or phone call 1 week following the initial treatment reminding them of their goals and steps for making changes.

##### 3 months

Participants in the early treatment group receive a further two sessions using the AIMhi Stay Strong app following the 3-month follow-up assessment. The two 20-min sessions occur 2–4 weeks apart and follow a similar format to the sessions received at baseline – reviewing family, strengths, worries, previous goals and sets new goals. A text message or phone call is sent 1 week following the initial treatment to remind participants of their goals and steps for making change and the time for the next session.

#### Contact control/delayed treatment- (CC/DT)

##### Baseline

Participants who are randomised to CC/DT receive 20 min of contact with the researcher using a culturally appropriate generic health App (i.e. The Hep B Story) at baseline, with a further 20-min session using the same app after 2–4 weeks. Session 1 of CC/DT goes through the structured Hep B App. Discussions specifically avoid review of family, strengths or individual goal setting. The participant interacts with the App with support from the researcher, and discussion focuses on navigation of the App and the App content. A ‘goal’ is agreed to talk to someone else in their family about the app content before the next session. A pictorial summary (utilising similar colours and images to the intervention summary) is given to the client. Session 2 reviews the information discussed in Session 1. This ensures each group receives the same contact time with researchers and interaction based on a structured App to aid participant blinding and to structure the control session. Participants receive a text message or phone call with a health tip linked with the health App in the intervening weeks.

##### 3 months

Participants in the CC/DT group then receive a 20-min MCP interview using the AIMhi Stay Strong App (on a tablet device) following their 3-month assessment, with one further 20-min session using the App within 2–4 weeks, following the format of the sessions received by the early treatment group at baseline.

#### Treatment as usual/delayed treatment (TAU/DT)

##### Baseline

Participants who are randomised to TAU/DT only receive the questionnaires and no other researcher intervention at baseline. Participants in this group receive only usual care from their renal service provider.

##### 3 months

After the 3-month assessment, participants in the TAU/DT group receive the MCP intervention, which is delivered using the same procedures as the Contact Control/DT group.

#### Fidelity of the intervention

The interventions are delivered by trained researchers who receive comprehensive training in delivery of the manualised MCP therapy and the CC activity through a two-day training workshop. The workshop is delivered by AIMhi trainers with reference to the AIMhi Stay Strong Planning Brief Treatment Manual [18], with booster sessions at 2-monthly intervals during the intervention phase. App usage data (e.g. number and type of goals and steps entered, amount of time spent on each page of the app etc) is reviewed for adherence to core MCP principles. Reviews of App data and ongoing booster sessions are used by the research team to provide regular feedback to researchers delivering treatment to redirect and adjust their mode of delivery as needed.

### Outcome measures

#### Primary outcome

##### Kessler distress scale (K-10)

K10 is a measure of psychological distress with strong links between high scores and anxiety and depression. K10 is one of the Australian Mental Health routine outcome measures and has been used in full and abbreviated forms in state and nation-wide Indigenous surveys [1, 19]. For the period July 2012–June 2013, the Australian Mental Health Outcomes and Classification Network (AMHOCN) reports mean K10 scores for ‘ambulatory’ patients with mood disorders (i.e. outpatients returning to community after being treated acutely) across Australia of 27.6 (SD = 8.5) upon return to community, 22.0 (SD = 8.5) at 91-day review, and 18.4 (7.6) upon discharge from outpatient service [20]. Considering these findings, and those of our previous study [9], a change/difference in K10 scores of 5 points is considered clinically significant. K10 is completed at baseline, 3 and 6 months follow up.

#### Secondary outcomes

##### Adapted patient health questionnaire (PHQ-9)

This tool assesses severity of depression and has shown diagnostic, criterion and construct validity [21]. It has been tested in Indigenous groups and adapted to include simplified response categories [22, 23] as well as specifically adapted for the central Australian context [24]. This culturally adapted version of the PHQ-9 is completed at baseline, 3 and 6 months follow up.

##### EuroQoL (EQ-5D) 5 level

The EQ-5D is a widely utilised multi-attribute utility instrument used for estimating utility weights for calculation of quality adjusted life years (QALYs). It is a self-report measure of quality of life in 5 domains (mobility, self-care, usual activities, pain and discomfort, anxiety and depression) and is used to calculate QALYs for the economic evaluation. Participants are supported to complete this at the time of the other assessments at baseline, 3 and 6 months follow up.

##### Healthcare resource use

The cost of delivering the intervention and total costs of health service usage (inpatient and outpatient) will be calculated. Costs will include the costs of dialysis, costs of inpatient hospitalisations and ED presentations and an estimation of outpatient health care use. Healthcare use will be based upon clinical file review.

### Sample size and power

We will recruit 156 participants over 15 months, allowing for up to 10% drop out at 6 months. With a SD of 8.5 for the baseline score and a sample of 62, 62 and 32 participants per arm, we will be able to detect a minimum difference between the group mean scores equal to 5 at 3 months with 90% power and an alpha of .05. This sample size will also give over 90% power to detect a minimum change in mean score within groups equal to 5. This calculation allows for 10% attrition. We consider a difference between the group mean scores of 5 to be clinically significant. A change of this magnitude (effect size = 0.6) suggests that the change would be positive for approximately 73% of the population.

### Statistical analyses

#### Demographic analyses

Demographic data will be tabulated and expressed as proportions and/or means of the selected characteristics by treatment group with the corresponding 95% Confidence Intervals (CI). Differences between groups will be assessed by the normal test for comparisons of means and χ^2^ tests for comparison of proportions.

#### Primary endpoint analyses

Continuous outcome measures will be compared between the three groups using a linear mixed effect model, with transformations applied to the outcome measure if not normally distributed. The mixed effect models with random effect intercept will allow for correlation between measurements of K10 scores taken at three points in time within the same subject, and will estimate the effect of the interventions at 3 and 6 months. All analyses will be conducted on an intention-to-treat basis. Preliminary analyses will check for group differences at baseline and control for them statistically if necessary.

#### Secondary endpoint analyses

The economic evaluation takes the perspective of the healthcare funder, including health outcomes based on the primary outcome and QALYs gained. As well as the health care costs outlined above, data is collected on the cost to deliver the intervention program (staff costs, training, capital costs and consumables). Using the mean costs and health outcomes in each trial arm, the incremental cost per 1) extra patient achieving a clinically meaningful improvement in the Kessler Distress Scale and 2) QALYs gained of the early treatment group compared with delayed treatment groups will be calculated; results will be plotted on a cost-effectiveness plane. Bootstrapping will be used to estimate a distribution around costs and health outcomes, and to calculate the confidence intervals around the incremental cost-effectiveness ratios. One-way sensitivity analyses will be conducted around key variables, and a probabilistic sensitivity analysis will be conducted to estimate the joint uncertainty in all parameters. A cost effectiveness acceptability curve will be plotted providing information about the probability that the intervention is cost effective, given willingness to pay for each additional QALY gained.

## Discussion

This project is expected to contribute much-needed evidence of effectiveness of wellbeing interventions for Indigenous people and demonstrate the benefits of providing such interventions to those with ESKD. It is the first randomised controlled trial to address both chronic disease and mental health in an Indigenous chronic kidney disease (CKD) population. It builds on an existing program of mental health research with demonstrated efficacy (AIMhi) to test the newly developed electronic motivational care planning therapy – the AIMhi Stay Strong App, for improving psychological distress, depressive symptoms, quality of life and adherence to dialysis among Indigenous ESKD patients. Knowledge translation strategies include plans to conduct two implementation workshops with consumers and stakeholders following data collection and analysis to explore strategies for implementation. Results will also be communicated through publications, culturally adapted resources including posters and flyers. By working collaboratively with existing service providers, the project should improve their capacity to intervene early and translate the research outcomes into sustained clinical practice, leading to improved access to treatment and better clinical outcomes for an extremely vulnerable population.

## Trial status

Protocol version number 2.3. Recruitment began February 2017 and is expected to be completed by February 2019.

## Abbreviations

AIMhi: Aboriginal and Islander Mental Health Initiative
AMHW: Aboriginal mental health worker
CC/DT: Contact Control/Delayed treatment
CI: Confidence interval
CKD: Chronic kidney disease
EQ5D: EuroQoL
ESKD: End stage kidney disease
K10: Kessler Psychological Distress Scale
MCP: Motivational care planning
NT: Northern Territory
PHQ-9: Patient Health Questionnaire
QALY: Quality adjusted life years
QoL: Quality of Life
RCT: Randomised controlled trial
TAU/DT: Treatment as usual/Delayed treatment
